# Neural synchrony in mother–child conversation: Exploring the role of conversation patterns

**DOI:** 10.1093/scan/nsaa079

**Published:** 2020-06-15

**Authors:** Trinh Nguyen, Hanna Schleihauf, Ezgi Kayhan, Daniel Matthes, Pascal Vrtička, Stefanie Hoehl

**Affiliations:** Department of Developmental and Educational Psychology, Faculty of Psychology, University of Vienna, Vienna 1010, Austria; Cognitive Ethology Laboratory, German Primate Center—Leibniz Institute for Primate Research, Göttingen 37077, Germany; Department for Primate Cognition, Georg-August-University Göttingen, Göttingen 37073, Germany; Department of Psychology, Social Origins Lab, University of California, Berkeley, CA 94720-1650, USA; Department of Developmental Psychology, University of Potsdam, Potsdam 14476, Germany; Max Planck Institute for Human Cognitive and Brain Sciences, Leipzig 04103, Germany; Max Planck Institute for Human Cognitive and Brain Sciences, Leipzig 04103, Germany; Max Planck Institute for Human Cognitive and Brain Sciences, Leipzig 04103, Germany; Department of Psychology, University of Essex, Wivenhoe Park, Colchester CO4 3SQ, UK; Department of Developmental and Educational Psychology, Faculty of Psychology, University of Vienna, Vienna 1010, Austria; Max Planck Institute for Human Cognitive and Brain Sciences, Leipzig 04103, Germany

**Keywords:** turn-taking, mother–child interaction, functional near-infrared spectroscopy, neural synchrony, conversation, hyperscanning

## Abstract

Conversations are an essential form of communication in daily family life. Specific patterns of caregiver–child conversations have been linked to children’s socio-cognitive development and child-relationship quality beyond the immediate family environment. Recently, interpersonal neural synchronization has been proposed as a neural mechanism supporting conversation. Here, we present a functional near-infrared spectroscopy (fNIRS) hyperscanning study looking at the temporal dynamics of neural synchrony during mother–child conversation. Preschoolers (20 boys and 20 girls, *M* age 5;07 years) and their mothers (*M* age 36.37 years) were tested simultaneously with fNIRS hyperscanning while engaging in a free verbal conversation lasting for 4 min. Neural synchrony (using wavelet transform coherence analysis) was assessed over time. Furthermore, each conversational turn was coded for conversation patterns comprising turn-taking, relevance, contingency and intrusiveness. Results from linear mixed-effects modeling revealed that turn-taking, but not relevance, contingency or intrusiveness predicted neural synchronization during the conversation over time. Results are discussed to point out possible variables affecting parent–child conversation quality and the potential functional role of interpersonal neural synchronization for parent–child conversation.

## Introduction

Preschool age is a critical time for children as they start to expand their daily interactions with family members and establish peer relationships outside the immediate family context (Harrist and Waugh, 2002). In this transitional phase, communicative abilities play a crucial role. Although interpersonal synchronization processes have recently been functionally implicated in successful communication and information transfer between adults (e.g. Schippers *et al.*, 2010; Hasson *et al.*, 2012), we currently know little about their role in child development. Here, we explore whether variables related to conversation patterns are associated with interpersonal neural and behavioral synchronization between mothers and their preschool-age children.

The ability to engage in and uphold verbal conversations becomes more sophisticated as children acquire language and advance in their social and cognitive competencies during preschool years (Black and Logan, 1995). Conversations are a special form of speech exchange as they are ‘managed by the participants, turn by turn, in terms of who speaks when, for how long and about what’ (Wilson and Wilson, 2005). The coordination needed to engage in and maintain a conversation is highly complex and presupposes multifaceted social processes, such as joint social attention and temporal contiguity (Romeo *et al.*, 2018). Previous studies reported that if such communicative patterns are lacking during parent–child dyadic conversations—i.e. the dyad is unable to coordinate and does not exhibit verbal turn-taking—atypical language development and the risk for developmental disorders more generally may arise in children (Moseley, 1990; Malin *et al.*, 2011). Furthermore, intervention studies indicate that the promotion of turn-taking leads to higher quality social interactions (Stanton-Chapman and Snell, 2011). The neural mechanisms supporting such central qualities in parent–child interaction and especially conversation, however, have only been sparsely investigated so far (Nguyen *et al.*, 2020). We propose neural synchronization as a neurobiological underpinning to successful communication and particularly turn-taking during parent–child conversations.

### Interpersonal neural synchrony

Communication between individuals, and more specifically turn-taking, has recently been linked to interpersonal neural synchronization (Stephens *et al.*, 2010; Hasson *et al.*, 2012). Interpersonal neural synchronization generally refers to the mutual temporal alignment of behavioral, neural and physiological activity (i.e. bio-behavioral synchrony) between two or more individuals (Hoehl, Fairhurst & Schirmer, in press). The neural aspect of interpersonal synchronization, in turn, is defined as the temporal coordination of concurrent rhythmic brain activities between individuals (Uhlhaas *et al.*, 2009). Finally, turn-taking involves a highly organized behavioral structure and timing that allows the speaker and listener to perceive and act upon cues provided during communication. For both conversation partners to adopt this kind of precision in mutual timing, Wilson and Wilson (2005) suggest that endogenous oscillators in the brain are involved. These neural oscillators are made of groups of neurons that collectively show periodic activity and are implicated in timing-related cognitive processes. During verbal communication, neural oscillators in one conversation partner both influence and adapt to the oscillators of the other partner(s) so that a dyad (or a larger group) can become mutually entrained based on each person’s speech production (e.g. temporal regularities in syllabic and word boundaries). Entrainment of such cyclic behavior has also been shown in breathing patterns of conversation partners (McFarland, 2001). With the advancement of ‘hyperscanning,’ that is simultaneously measuring brain activity of two (or more) participants, neural synchrony has been proposed as evidence for the mutual alignment of endogenous neural oscillators during interpersonal communication (for reviews see Dumas *et al.*, 2011; Hasson *et al.*, 2012).

In adults, hyperscanning has been applied in dyadic and group conversation contexts (e.g. Jiang *et al*., 2012, 2015, 2016; Spiegelhalder *et al.*, 2014; Nozawa *et al.*, 2016; Pérez *et al.*, 2019). In the study by Jiang *et al.* (2012), face-to-face dialogs were contrasted with face-to-face monologs and back-to-back dialogs and monologs. Their findings showed neural synchronization in the left inferior frontal cortex exclusively during face-to-face dialogs between dyads in comparison to all other conditions. These results first highlighted the importance of multi-sensory input during live social interactions for neural synchronization. Additional studies went on to identify factors facilitating neural synchronization during verbal communication, such as leader emergence (Jiang *et al.*, 2015), eye contact (Jiang *et al.*, 2016) and attention towards a speaker (Dai *et al.*, 2018). Whereas some researchers highlighted the role of turn-taking for information exchange and neural synchronization (Pérez *et al.*, 2019), others suggested that synchronized oscillatory patterns would underlie successful turn-taking (Wilson and Wilson, 2005). Although the above studies point towards a relationship between neural synchrony and turn-taking, the exact nature of the interaction between turn-taking and interpersonal neural synchrony is largely unknown.

A growing body of hyperscanning studies examining adult–child dyads focused on either task-based interaction (Reindl *et al.*, 2018; Miller *et al.*, 2019; Nguyen *et al.*, 2020) or free-play interactions with non-verbal infants (Piazza *et al.*, 2020). Due to the context provided by the tasks or the age of assessed children, mostly non-verbal factors associated with neural synchronization have been explored thus far. In two of the available studies, a more naturalistic interaction allowed the additional examination of interaction quality in association with neural synchrony (Quiñones-Camacho *et al.*, 2019; Nguyen *et al.*, 2020). The findings showed that in preschool child–parent dyads, behavioral reciprocity during joint problem solving was correlated with higher neural synchronization. Behavioral reciprocity may thus also play a role for interpersonal neural synchronization in parent–child verbal conversations. Yet, little is known about which verbal communicative patterns influence neural synchronization in parent–child conversations specifically.

### Conversation patterns conducive to information exchange

Behavioral research suggests that interactions featuring contingent turn-taking and responsiveness are more effective for social learning (see, for example, Begus and Southgate, 2018). From infancy on, turn-taking in mother–infant ‘proto-conversations’ has been implicated in language processing and acquisition (Levinson, 2016). Turn-taking also gives way to coordination in behavior, particularly reciprocity (Leonardi *et al.*, 2016). As the child’s sensitivity to vocal behavior grows, overlap of speech in dialog has been reported to be rather low and to even decrease over time. To uphold the chain of turns, both the quantity of contingent responses (Jaffe *et al.*, 2001) and the quality in terms of how a child is responded to appear to matter (Murray *et al.*, 2016). Furthermore, school-aged children not only talk in alternating turns but also participate in relevant and contingent discourses with their parents (Black and Logan, 1995). Interestingly, higher occurrences of these conversation patterns were linked to child likability among peers. This comes to show that parent–child communication patterns at preschool age may affect children’s social competence beyond language learning (Levinson, 2016).

### Temporal dynamics of interpersonal coordination

In general, there are many fluctuations throughout parent–child interactions as the dyad may not always interact in a coordinated manner (Tronick and Cohn, 1989). Research in healthy samples showed that mother–infant and mother–toddler interactions are composed of periods involving high occurrence of reciprocity, mutual gaze and/or affect mirroring, characteristic for high-interaction quality. Such periods were found to be interspersed with brief ruptures of miscoordination that were successfully repaired so that the dyad could return to a coordinative state. Critically, higher fluctuations in behavioral and physiological coordination have been associated with families at risk for child maltreatment (Skowron and Reinemann, 2005; Giuliano *et al.*, 2015). Much less is known, however, regarding the exact temporal dynamics of interpersonal synchrony on a neural level in parent–child interactions, because most of the extant research has focused on averaged synchrony values over the whole length of given experimental conditions (e.g. Reindl *et al.*, 2018). There is some available data from adult dyads by Mayseless *et al.* (2019), who examined the temporal dynamics of neural synchrony during a creative problem-solving task. They showed that neural synchrony decreased throughout the task and that such decrease in neural synchrony was linked to an increase in behavioral cooperation. These data show that neural synchrony may change over the course of a longer task and that such variation may associate with behavioral and/or communicative patterns—also during mother–child conversation. We thus argue that investigating dynamic changes of interpersonal neural synchrony in parent–child conversations will provide additional information towards generating a neurobiological model of parent–child interactions, as variations reflecting factors that affect neural synchrony might otherwise not be captured.

### Current study

In the present study, we examined a free verbal conversation between mothers and their preschool children to identify conversation patterns associated with neural synchronization. We expected the naturalistic face-to-face situation to allow for dyadic differences in conversation patterns between mother and child dyads to emerge. Individual brain activity of mothers and children was simultaneously measured using functional near-infrared spectroscopy (fNIRS) by focusing on frontal and temporo-parietal regions and subsequently assessed for interpersonal temporal alignment (i.e. interpersonal neural synchrony) by means of wavelet transform coherence (WTC) analysis. Chosen regions of interest (ROIs) are known to be involved in social-cognitive processes during live interaction (Redcay and Schilbach, 2019) and have previously been shown to display increased neural synchronization during face-to-face conversation (Jiang *et al.*, 2012).

We aimed to identify relevant conversation patterns, such as turn-taking, and to investigate their associations with interpersonal neural synchrony. Based on previous work (e.g. Jiang *et al.*, 2012), we hypothesized that turn-taking would be positively associated with neural synchronization during mother–child verbal conversation. In addition, we were interested in the dynamic time-course of neural synchrony during the mother–child conversation. A finer resolution of the time dynamics of interpersonal neural synchrony during a conversation can add to the understanding of such complex social interactions. We, therefore, hypothesized that neural synchrony would change over time in association with the frequency of turn-taking shown during the mother–child conversation. When considering turn-taking features during verbal exchanges where intervals between turns mostly range from 200 to 700 ms (Gratier *et al.*, 2015), it is reasonable to assume that intrusive and non-responsive behavior would relate to attenuated neural synchrony. Next to the hypothesized role of turn-taking for neural synchronization, we were probing the relation between conversational relevance as well as contingency and interpersonal neural synchronization, as these variables were shown to be related to turn-taking patterns (Fine, 1978).

## Material and methods

### Participants

Forty mothers (mean age 36.37 years; s.d. = 4.51 years; range = 28–47 years) and their preschool children (20 boys and 20 girls; mean age 5;07 years; s.d. = 0;04 years; range = 4;11–6;01) were included in the present study. Out of initially, 46 recruited mother–child pairs, six were excluded due to technical problems or self-reported tiredness/fussiness. All included dyads took part in the condition for the whole of the 4 min. Fifty-eight percent of mothers graduated with a university degree, while the remaining mothers graduated from vocational school. Each mother–child pair was biologically related. Participants were recruited from a pre-existing database of volunteers and mothers gave written informed consent for both themselves and their children before participating in the study. We screened for psychiatric/neurological disorders of mothers and children for developmental delay according to mother’s self- and parent-report as part of their application to be included in the database. The study procedure was paused as soon as the child or the parent showed any sign of discomfort. Procedures were approved by the local ethics committee and participation was remunerated.

### Experimental procedure

**Fig. 1:**
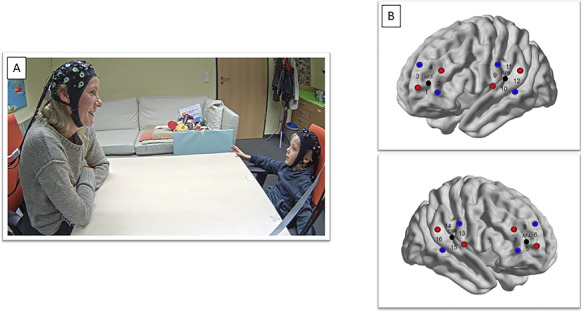
(A) Study set-up during the free verbal conversation task. (B) Cap configuration. Red circles mark sources and blue circles mark detectors. Numbers (1–16) mark measurement channels between sources and detectors and black circles mark EEG 10–20 channel positions for orientation. The top graphic shows the left hemisphere and the bottom graphic shows the right hemisphere.

During the experiment, mothers and their children sat face-to-face (see Figure 1A), separated by a table. After performing two cooperative and two individual problem-solving task conditions in a naturalistic setting with a tangram puzzle (~12 min) that were unrelated to the present investigation (reported in Nguyen *et al.*, 2020), mothers and children were instructed to engage in a free verbal conversation for 4 min. The instruction is detailed in the Supplementary Section S1. The complete procedure was video recorded from three different angles.

### fNIRS data acquisition

We used a NIRScout 8-16 (NIRx Medizintechnik GmbH, Germany) Optical Topography system to record oxy-hemoglobin (HbO) and deoxy-hemoglobin (HbR) concentration changes for each participant. The four 2 x 2 probe sets were attached to an EEG cap with 10–20 configurations. The standard electrode locations allowed us to place the probes more precisely, as the probes over the left and right dorsolateral prefrontal cortex (dlPFC) surrounded AF3 and AF4 and the probes on the left and right temporo-parietal junction (TPJ) surrounded CP5 and CP6 (see Figure 1). ROIs were based on previous fNIRS hyperscanning work involving verbal communication (Jiang *et al.*, 2012, 2015). In each probe set, eight sources and eight detectors were positioned, which resulted in 16 measurement channels with equal distances of 3 cm between the optodes per participant. The absorption of near-infrared light was measured at the wavelengths of 760 and 850 mm and the sampling frequency was 7.81 Hz.

### fNIRS data analysis

Data were pre-processed using MATLAB-based functions derived from Homer2 (Huppert *et al*., 2009). Raw optical density data were motion-corrected with a wavelet-based algorithm (Molavi and Dumont, 2012). Corrected data were then visually inspected during an initial quality check procedure. All channels that did not show a clear heart band were removed, which resulted in 93.4% of the channels to be included in further analyses. Data were then band-pass filtered with low- and high-pass parameters of 0.5 and 0.01 using a second-order Butterworth filter with a slope of 12 dB per octave (Baker *et al.*, 2016). Next, the filtered data were converted to HbO and HbR values based on modified Beer-Lambert Law with differential path length factors of 6 for adults and 5.5 for children. Based on previous hyperscanning studies, statistical analyses were focused on HbO values, which were reported to be more sensitive to changes in the regional cerebral blood flow (Hoshi, 2007). However, all analyses were repeated for HbR values (reported in Supplementary Section S4).

**Fig. 2:**
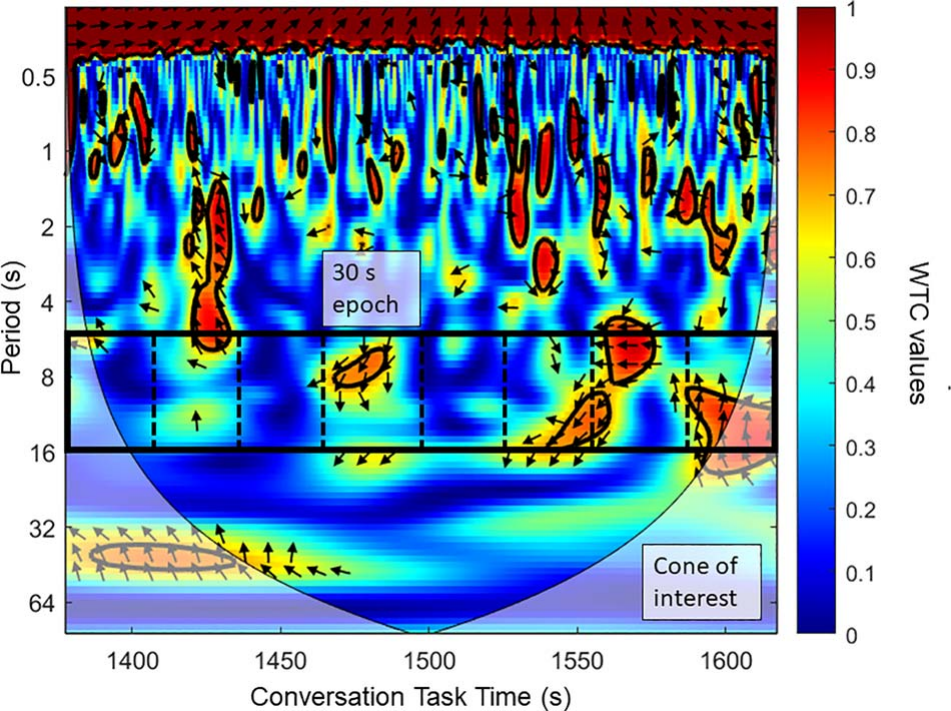
WTC values were calculated by averaging over the frequency range of interest (6–16 period seconds—~0.06 Hz—0.15 Hz—on the *y*-axis) for the entire experimental procedure and epochs of 30 s over the 4 min of the conversation condition (indicated by one square along the *x*-axis [time in seconds]). Coherence values ranged from 0 to 1 (as indicated by the color bar) and all coherence values outside the cone of interest demarcation were excluded from analyses.

Subsequently, neural synchrony was calculated with WTC analysis using the cross wavelet and wavelet coherence toolbox (for more information, see Grinsted *et al.*, 2004; Chang and Glover, 2010). WTC was used to assess the relation between the individual fNIRS time series in each dyad and each channel as a function of frequency and time. Based on earlier literature (e.g. Jiang *et al.*, 2015), visual inspection and spectral analyses identified the frequency band of 0.06–0.15 Hz (corresponding to ~6–16 s) as related to the free verbal conversation. This frequency band did not comprise high- and low-frequency noise—such as respiration (~0.2–0.3 Hz) and cardiac pulsation (~1 Hz). Furthermore, coherence values outside the cone of influence were excluded in the WTC analysis. Average neural coherence (i.e. neural synchrony) was then calculated for 30-s epochs in each channel, which resulted in 8 (epochs) × 16 (channels) coherence values for each dyad (see Figure 2). Epoch length was defined by the minimal time needed to estimate an appropriate coherence value for the indicated frequency range.

### Communication pattern coding

Communication codes were adapted from Black and Logan (1995). This involved coding of communicative reciprocity of mother–child dyads (operationalized through turn-taking), the thematic fit of the utterance (relevance), responding to questions or requests (contingency), and interruptions, overlaps, as well as intrusiveness (as further described in Table 1). Utterances were coded in Mangold INTERACT by trained graduate students. Utterances were chunked into turns that were defined as ‘one person’s speech bounded by pauses or by the speech of another person’ (Garvey and Kramer, 1989) and transition between turns marked by minimal gaps (ranging from a few milliseconds to 3000 ms) and minimal overlap (Gratier *et al.*, 2015). Twenty percent of conversations were double coded by two graduate students and inter-rater reliability was calculated by intraclass correlations (ICC) for each communication code. ICC ranged between 0.70 and 0.99 and averaged at 0.84, therefore showing high reliability between coders. A score for each category was then calculated by building a composite score of relevant subcategories, which were all equally weighted. This approach in data reduction resulted in one value each for turn-taking, relevance, contingency and intrusiveness per dyad.

**Table 1:** Conversation patterns divided into categories, sub-categories, examples and inter-rater reliability

Category	Sub-category	Example	ICC
Turn-taking	Alternating turns: An utterance follows a turn by the other speaker	Child: ‘It was such a fun game’ Mother: ‘Yes, what shape did you like building the most?’	0.90
	Long turns: One speaker follows up with another utterance after an utterance	Child: ‘It was such a fun game. There were all these shapes and colors and I couldn’t decide what to begin with.’	0.70
Relevance	Relevant turns: The utterance shares the thematic content with the preceding initiation or response	Child: ‘I liked the rocket the most’ Mother: ‘Me, too. The rocket had an interesting shape’	0.92
	Irrelevant turns: The utterance lacks shared thematic content with the preceding utterance	Child: ‘I liked the rocket the most’; Mother: ‘The world map is so colorful’	0.81
Contingency	Contingent utterances: A turn provides the requested information to the previous utterance or the conversation partner performs requested activity	Mother: ‘Do you want to start with the puzzle?’ Child: ‘Ok’ and both start to play	0.99
	Noncontingent utterances: A speaker fails to respond to the previous request or question	Mother: ‘Do you want to start with the puzzle?’ Child: ‘I don’t know where this shape has to go.’	0.81
Intrusiveness	Turns that fail to leave time for a response: A speaker makes a request and fails to leave time for the other person to respond	Mother: ‘Do you want to play with the puzzle? Oh, are you looking forward to this weekend?’	0.79
	Turns that interrupt: A speaker begins a turn before the other person has finished his or her turn	Mother: ‘We can start with -?’; Child: ‘I really like this shape’	0.96
	Simultaneous turns: Two speakers attempt to speak at the same time	-	0.72

### Turn and overlap duration coding

Next to the coding of communication features, we assessed turn and overlap duration between utterances. Turn duration was assessed by pauses indicating switches between speakers that ranged from <50 ms to a maximum duration of 3000 ms. Whenever one interaction partner vocalized over the other partner, an overlap for the duration was coded. Twenty percent of conversations were double coded by two graduate students and inter-rater reliability was calculated with weighted kappa. Kappa for turn and overlap duration ranged between 0.81 and 0.97 and averaged at 0.91, therefore showing very high reliability between coders.

### Statistical analysis

Generalized linear mixed models (GLMMs) were fitted with the package ‘glmmTMB’ (Magnusson *et al.*, 2017) extended by custom functions (personal communication with Mundry, 2018) in R Studio (RStudio Team, 2015). For neural synchrony analyses, WTC values were entered as the response variable assuming a beta distribution (because all values of a beta distribution are bound between 0 and 1, such as the values of the WTC). All continuous predictor variables were *z*-standardized, and distribution of residuals was visually inspected for each model. Models were estimated using maximum likelihood. Model fit was compared using a likelihood ratio test (Dobson, 2002). To further test significant interaction effects, the function ‘emtrends’ from the package ‘emmeans’ (Lenth, 2019) was used.

Instead of including ROIs as a fixed effect predictor in the above-mentioned GLMMs, separate models for each region of interest were conducted, which are reported in the Supplementary Section S3. This approach was chosen because optical properties in different regions are suggested to vary systematically and therefore introduce a bias in the analysis. A full random effects structure for all models was assumed and thus random intercepts for dyad, and a random slope for each added fixed and interaction effect were included. The correlation of the random slopes with the random intercept for dyads was removed (as indicated by *||* in the model formulae in Supplementary Table S2) to help with convergence issues. The resulting random effects structure is shown in the model formulae. All model outcomes for models 1–7 can be found in Supplementary Table S2.

To rule out effects due to spurious correlation, we conducted a random pair analysis (Leong *et al.*, 2017; Piazza *et al.*, 2020). For each original mother–child pair, fNIRS time-courses of children were paired with time-courses of 1000 random mothers. Again, the WTC means were obtained across eight 30 s epochs and the frequency band of interest for each channel (8 epochs × 16 coherence values × 1000 random pairs). Coherence values of original dyads were then tested against the average of randomized pair coherences (considered a threshold for significant synchronization) in each ROI and over time by comparing coherence values using GLMM (see Supplementary Section S3 for separate GLMM in each ROI). This control procedure was repeated using phase randomization as a stricter control analysis (see Supplementary Section S2).

## Results

In the present study, we investigated whether mother–child dyads show neural synchronization in temporo-parietal and dorsolateral areas when the dyads engage in a free verbal conversation. Furthermore, we explored the temporal dynamics of neural synchronization throughout the observation and particularly whether certain conversation patterns are associated with increases or decreases in neural synchronization. Specifically, we probed the role of turn-taking, relevance, and intrusiveness for interpersonal neural synchrony in mother–child conversations.

### Neural synchrony during conversation

First, we conducted control analyses to determine whether mothers and their children showed higher neural synchronization during the conversation in comparison to randomly paired surrogate dyads (random-pair analysis). WTC was entered as the response variable and the random intercept of each child (indicated by the variable ID) was included in the null model. We assumed dependency between original and random pairs of children and mothers, even though it is important to note, that original and random pairs are neither fully dependent nor independent. In line with previous results in adult–child dyads (Leong *et al.*, 2017), original mother–child dyads showed increased neural synchronization, *M*(s.d.) = 0.322(0.002), in comparison to neural synchrony values of randomly paired dyads, *M*(s.d.) = 0.315(0.002), as adding the fixed effect and random slope of pairing resulted in a significant improvement of model fit, *χ*^2^(3) = 19.71, *P* < 0.001. Next, we assessed how neural synchrony of original and random dyads behaved over time. When adding the fixed effect and random slope of time in each dyad, the model fit improved significantly, χ^2^(3) = 23.56, *P* < 0.001. Neural synchrony changed in both original and random pairings over time. The next model further included the interaction effect between pairing and time, as well as the random slope of the interaction and shows that the dynamic changes of neural synchrony over time differ in original and random pairings, χ^2^(3) = 16.12, *P* < 0.001. Comparing the trend in the change of the original coherence (trend = 0.006, SE = 0.003, 95% CI = [−0.004 0.017]) over time to the change in the random coherence (trend = −0.007, SE = 0.003, 95% CI = [−0.010–0.002]), showed that while original neural synchrony increased over time, random neural synchrony decreased over time. To conclude, original pairs not only showed significantly higher neural synchrony than random pairs, but also showed a positive, instead of a negative trajectory over the course of the conversation.

**Fig. 3:**
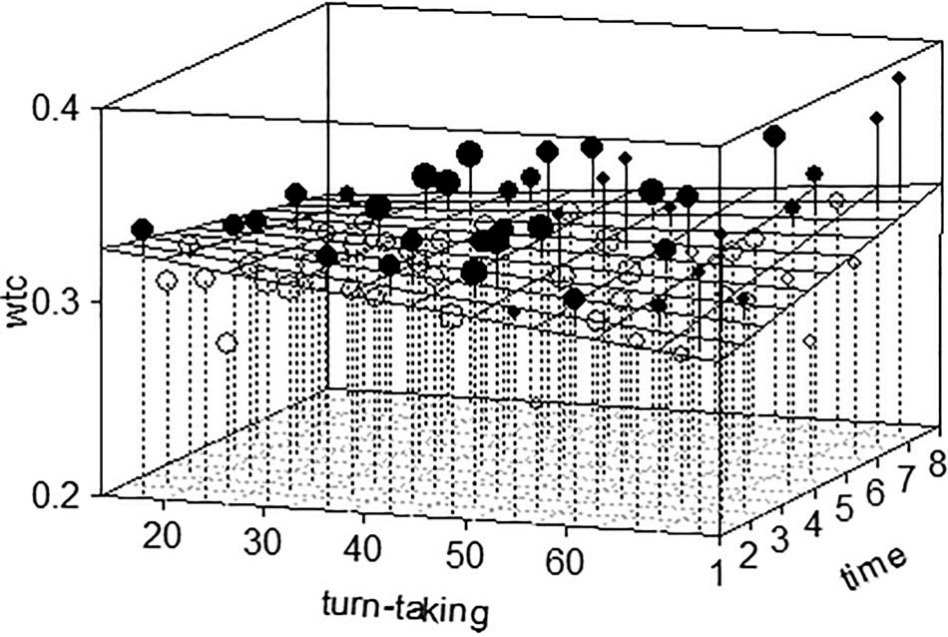
3D-plot depicting the interaction between the number of turns taken (*x*-axis) and time in epochs of 30 s (*z*-axis) on neural synchrony (WTC; *y*-axis). The horizontal plane depicts estimates of neural synchrony extracted from the linear mixed-effects modeling. Black and white dots show the observed value of each dyad in each channel either above their model estimate (black) or below their model estimate (white). Larger dots indicate that a higher number of observations were summarized within the dot, while smaller dots indicate fewer observations. Overall, the plot depicts that more turn-taking was linked to higher neural synchronization towards the end of the conversation.

Next, we examined the role of turn-taking in neural synchronization over the 4 min of free verbal conversation. The null model comprised WTC as the dependent variable and a random intercept for each dyad. To test for the main effect of turn-taking, we first entered verbal turn-taking as a fixed-effect predictor and a random slope (Model 1). Model 1 showed improved model fit in comparison to the null model und thus depicts that turn-taking patterns were significantly related to interpersonal neural synchrony during the conversation, *χ*^2^(2) = 6.83, *P* = 0.033. Higher amounts of turn-taking were associated with higher neural synchronization between mother and child. When the fixed effect and random slope of time was added to the model (Model 2), the model fit improved significantly, *χ*^2^(2) = 12.45, *P* = 0.006. The estimates showed that the dyads seem to show increases in neural synchrony over time. Subsequently, we added the interaction effect as well as the random slope of the interaction between turn-taking and time (Model 3). The model fit improved significantly in comparison to Model 2, *χ*^2^(2) = 19.54, *P* < 0.001 (Figure 3). To further investigate the interaction effect between turn-taking and time, we went on to dichotomize our variable time into early epochs (1–4) and late epochs (5–8). Follow-up contrasts showed that the trend for turn-taking in later epochs, trend = 0.009, s.d. = 0.004, 95% CI = [0.008 0.069], was marginally higher than in earlier epochs, trend = 0.001, s.d. = 0.004, 95% CI = [−0.029 0.039]. Hence, there was an indication for higher turn-taking to relate to higher neural synchrony in later epochs. We then also split turn-taking into two groups (split by the median) to further contrast the high and low turn-taking groups in earlier and later epochs, respectively. Using emmeans and pairwise contrasts, we find that the group with higher amounts of turn-taking showed an increase in neural synchrony in later in comparison to earlier epochs, estimate = −0.017, s.d. = 0.006, 95% CI = [−0.031–0.002] (see Supplementary Figure S1). The group with lower amounts of turn-taking, however, showed no differences in neural synchrony between earlier and later epochs, estimate = −0.003, s.d. = 0.005, 95% CI = [−0.017 0.010]. The results thus underscore the association between high amounts of turn-taking and increases in neural synchrony over the course of the conversation, while less turn-taking was associated with no significant changes in levels of neural synchrony over time.

Next to turn-taking, we probed the association of neural synchrony and further conversation patterns. Hence, we added turn duration as a fixed effect and a random slope (Model 4) to previous fixed and random effects in Model 3, which did not improve the model fit, *P* = 0.301. Subsequently, relevance (Model 5), intrusiveness (Model 6) or contingency (Model 7) were all added as fixed effects and random slopes to Model 3, but did not significantly improve the model fit, *P* = 0.417, *P* = 0.832 and *P* = 0.648, respectively*.* Further information on linear mixed effect parameters is included in the supplements.

## Discussion

Learning how to communicate is key to exchange information with other people and connect with them (Papousek and Papousek, 1992; McComb and Semple, 2005). In adults, successful communication is known to be related to interpersonal synchronization in brain activation (Stephens *et al.*, 2010). Here, we investigated how affiliated dyads—mothers and their children—communicate with each other and how their communicative patterns relate to synchronous brain activation patterns. We particularly focused on verbal turn-taking and showed that during this specific process of interpersonal coordination, a higher number of turns between mothers and children was related to higher neural synchrony in frontal and temporo-parietal areas (Meltzoff and Decety, 2003; Redcay and Schilbach, 2019), which increased over the course of the conversation. These results represent an essential step in understanding the temporal dynamics of neurobehavioral synchrony in caregiver–child conversations with natural variability.

We found that mothers and children showed above chance-level neural synchronization in HbO in temporo-parietal (temporo-parietal junction—TPJ) and prefrontal (dorsolateral prefrontal cortex—DLPFC) areas during a free verbal conversation. Activation in temporo-parietal areas and particularly the TPJ is associated with the mentalizing system, which is implicated in both children and adults when trying to understand others’ mental states (Frith and Frith, 2006; Koster-Hale and Saxe, 2013). A recently presented framework on interactional synchrony by Hoehl *et al.* (2020) posits that the human brain constantly tracks temporal regularities in sensory input (e.g. auditory rhythms) employing striato-cortical loops. However, alignment to these rhythms depends on a range of stimulus properties and their socio-emotional meaning, which is computed in temporoparietal regions, including TPJ. The dorsolateral prefrontal cortex, on the other hand, is functionally related to the cognitive control system and recruited during tasks that involve top–down control over cognitive and emotional processes in social contexts (Grossmann, 2013; Balconi and Pagani, 2015). Both the mentalizing and cognitive control systems have been implicated in neural synchronization processes in various contexts of interactions, such as conversation, cooperative problem-solving and joint action in adult dyads (Jiang *et al.*, 2016; Liu *et al.*, 2016). However, the present study is the first to date to find these regions associated with neural synchronization during a mother–child verbal conversation. Still, the basic function of neural synchrony in areas involving the mentalizing and cognitive control system during conversation needs to be clarified in further studies. One interesting path for future research in adult dyads would be to experimentally manipulate interpersonal neural synchrony in these brain regions and assess the effects on social cognition (see Novembre *et al*., 2017).

Next, we were interested in probing time-dependent changes of neural synchrony in relation to conversation patterns. Accordingly, we examined the temporal dynamics of neural synchrony during eight 30 s intervals of the 4-min-long conversation. In contrast to findings obtained in adult dyads (Mayseless *et al.*, 2019), we found that neural synchrony in temporo-parietal and lateral prefrontal regions increased throughout the conversation on average. This discrepancy could be a result of variation in interaction type-related factors as well as individual differences of participants. Firstly, while the interaction assessed during the adult study was a creative problem-solving task, participants in the present study engaged in a free verbal conversation. Second, temporal changes in neural synchrony could be affected by the type of interaction with free conversations constantly involving re-synchronization processes due to their high complexity (Richardson *et al.*, 2008). Third, the amount and effort of coordination could also differ as a function of the relationship quality between the two interaction partners, which could result in different temporal dynamics. Two previous studies employing a cooperative interaction task observed higher synchronization between affiliated dyads compared to non-affiliated stranger dyads (Pan *et al.*, 2017; Reindl *et al.*, 2018). Here, we tested mother–child dyads, who are closer and more accustomed to one another than strangers, and thus, may be faster to find their own mutual rhythm (Markova *et al*., 2019). In future studies, it will be important to investigate interindividual differences of the interacting dyads’ relationship, ideally including a range of variables also comprising parent–child attachment (see Leclère *et al*., 2014; Long *et al.*, 2020).

Critically, when we considered how often mothers and children took turns, higher neural synchrony was associated with higher turn-taking in later epochs of the conversation. Evidence from studying an individual’s entrainment to rhythmic auditory stimuli shows that neural signal alignment is a gradual process and sustains temporarily even after the stimulus is no longer present (Trapp *et al.*, 2018). We therefore suggest that interpersonal alignment of brain activity might assume a similar pattern in that the regularity of turn-taking in parent–child interactions could take effect further along the conversation instead of resulting in immediate alignment. Overall, increased interpersonal neural synchrony during parent–child conversation could reflect higher turn-taking quality, implying a high level of mutual attention and gradually increasing mutual adaptation.

Turn-taking and language development are tightly intertwined, as studies show that conversation patterns, such as turn-taking duration, indicate how fast children can understand a question while planning and initiating their response at the same time (Casillas *et al*., 2016). Especially contingent speech by parents, as in attuned turn-taking, helps infants to simplify the structure of speech and language and thus catalyze their language production (Goldstein *et al.*, 2003; Elmlinger *et al.*, 2019). What is more, turn-taking was shown to be implicated in heightened neural language processing (Romeo *et al.*, 2018): 4- to 6-year-old children who experience more conversational turns with adults showed greater left inferior frontal (Broca’s area) activation in individual neural measurements. Furthermore, the neural activation mediated the relation between children’s contingent language exposure and verbal skills. This finding highlights a potential future avenue of investigation into the role of neural synchrony for language development.

Other conversation patterns, such as cohesiveness of conversation indicated by relevance and contingency, were not associated with neural synchronization in the current study. The same was true for communicative intrusiveness. Although previous studies showed that both turn-taking and cohesiveness during parent–child conversations were critical and predictive for later language abilities (Roseberry *et al.*, 2014; Hirsh-Pasek *et al.*, 2015), we only found a link between turn-taking and neural synchrony during a free verbal conversation. Our predefined ROIs where we measured neural synchronization may not have captured such link to cohesiveness, as an influence of cohesiveness was previously observed, for example, in language-related areas such as the left inferior frontal regions (Romeo *et al.*, 2018). Future studies should explore whether neural synchrony in different regions could map onto different conversation patterns.

When we went on to explore the associations between communication patterns and interpersonal neural synchrony in HbR, we were not able to replicate our findings as for HbO synchrony, in contrast to an adult hyperscanning study (Pan *et al.*, 2017). The difference in results could be due to physiological differences between children and adults (Perlman *et al.*, 2014). The blood flow and oxygen metabolism coupling in children is suggested to differ from adults with concurrent increases in HbO and HbR found at times. Hence, further studies exploring both HbO and HbR neural synchrony in parent–child interactions are needed to decipher synchronization processes during early childhood across different age groups.

Our study had several limitations. First, fNIRS data were not measured during a control condition such as a resting period. We would argue, though, that resting phases are not an ideal control condition because changes in synchrony might rather be due to task-evoked changes in the autonomic nervous system (ANS) instead of in neural activity (Tachtsidis and Scholkmann, 2016). We therefore opted for a random pair analysis to control for changes in the ANS as well as other spurious correlations in the signal. In future studies, concurrent measurements of short channel regressors could further improve the signal. Next, due to the limited number of available optodes, we focused on cortical regions that appeared most relevant to social processes and were previously shown to be involved in neural synchronization during similar tasks. In further investigations as well as with the development of devices comprising more measurement channels, neural synchrony in language-related cortical areas could be examined (Zhao *et al.*, 2019).

## Conclusion

Our study shows that children and mothers synchronize their brain activity during natural verbal conversation and that neural synchronization increases over time when mother and child engage in more verbal turn-taking. This observed link between conversational turn-taking and neural synchronization opens up new possibilities to understand the potential functional role of neural synchronization during verbal exchanges. Future studies could explore the role of neural synchrony in language acquisition. Overall, our findings point towards neural synchrony as a potential neurobiological marker of successful coordination in mother–child conversation.

## Supplementary Material

nsaa079_Supp
