# Supplementary material for: Neural synchrony in mother–child conversation: Exploring the role of conversation patterns
Source: Soc Cogn Affect Neurosci. 2020 Jun 15;16(1-2):93–102. doi: 10.1093/scan/nsaa079 (PMC7812624; doi:10.1093/scan/nsaa079)
Supplement: nsaa079_Supp [file nsaa079_supp.zip › scan-19-424-File002_nsaa079.docx]

**Supplementary Information**

1. Instruction for the free verbal conversation

“As the last task for today, I would like you to have a conversation for the next four minutes. You are free to talk about whatever you like, this can include the task you just did together, whether you liked it, what was fun about it, but you are also free to talk about plans you have for today as well as the following days. Birthdays, past and future trips, your plans for the holidays are some of the possible topics. If you have any questions, feel free to ask me! Have fun and see you in a bit!”

1. Control analyses: Random pair and randomized-phase analysis

To further test for which regions reached significant neural, we calculated separate Generalized Linear Mixed Models for each ROI. WTC was entered as the response variable and the random effects structure included a random slope for pairing, time and their interaction in the random intercept for dyads. In the left dlPFC only the fixed effect of pairing increased the model fit, χ^2^(3)=4.16, *p*=.04, showing higher original neural synchrony in comparison to random synchrony. The effect of time and the interaction effect of time with paring, were not significant, *p*>.11. In the right dlPFC only the fixed effect of pairing marginally increased the model fit, χ^2^(3)=2.99, *p*=.08, again showing higher original neural synchrony in comparison to random synchrony. Time and the interaction effect with time did not improve the model fit, *p*>.17. In the left TPJ, both the fixed effect of pairing and the interaction effect of pairing and time improved the model fit, χ^2^(3)=22.97, *p*<.001, χ^2^(3)=20.93, *p*<.001, respectively. Neural synchrony of original dyads was higher than synchrony of random dyads. Furthermore, original dyads showed increases in neural synchrony over time (*trend*=0.016, *SE*=0.005), while random dyads showed decreased in neural synchrony over time (*trend*=-0.013, *SE*=0.005), *t*=4.57, *p*<.001. The fixed effect of time did, however, not explain any more variance in the data, *p*=.77. In the right TPJ, neither the effect of pairing nor of time explained more variance in the data, *p*>.24*.* Yet, the interaction between pairing and time significantly improved the model fit and depicted the same increasing neural synchrony in original dyads (*trend*=0.009, *SE*=0.006) and decreasing neural synchrony in random dyads (*trend*=-0.005, *SE*=0.005), *t*=2.30, *p*=.021. Overall, control analyses in individual regions reveal original neural synchrony values to be higher than neural synchrony in random pairings in the left dlPFC and left TPJ. Differences in the right dlPFC and right TPJ were either marginal or trajectories differed over time yet revealed no significant difference in the mean of synchrony values.

We further conducted a control permutation analysis with phase-shuffled time-series of each pair [(Lerner, Honey, Silbert, & Hasson, 2011)](https://www.zotero.org/google-docs/?eYeLBE). Fast Fourier transform was applied to the fNIRS time-course of mothers and the phase of the frequencies was subsequently randomized. An inverse Fourier transform was then applied to obtain a phase-shuffled time-course. Each dyads time-series was thus phase-randomized before submitted into the calculation of WTC. This procedure was repeated for 100 permutations, resulting in a distribution of phase randomized coherence values. The values were again averaged in each channel and epoch to estimate a coherence threshold and tested against original coherence values using GLMM. Replicating the results from the random pair analysis, surrogate dyads with phase-randomized time series showed less neural synchronization in comparison to original dyads, *χ*^2^(3)=10.47, *p*=.001. Neural synchrony changed over time in both dyads, *χ*^2^(3)=25.87, *p*<.001. However, the addition of the interaction effect between pairing and time depicted the change in time to not differ between dyads, χ^2^(3)=0.00, *p*=.926. This comes to show, that phase randomization as a control analyses, which preserves the mean and autocorrelation of the signal, still reveals the difference in mean between original and phase-randomized coherence.

1. Separate analyses for each region of interest

Optical properties in different regions are suggested to vary systematically and introduce a bias in the analysis. We, therefore, conducted additional exploratory analysis on each of the regions of interest and henceforth refitted Model 1-6 to the four channels in each region. In the left dorsolateral prefrontal cortex, Model 2 adding time and Model 3 adding the interaction between turn-taking and time significantly improved the fit in comparison to earlier models, *χ*^2^(2)=12.73, *p*=.001, *χ*^2^(2)=13.58, *p*=.001, respectively. Next to the significant fixed effect of time, which shows increases in neural synchrony over time, the findings reveal an interaction effect of turn-taking and time. Contrasts between earlier and later epochs reveal a positive relation between turn-taking and neural synchrony in the later epochs, *trend*=0.006, *SE*=0.008, 95% *CI=*[-0.011 0.023], which was higher than in earlier epochs, *trend*=-0.007, *SE*=0.009, 95% *CI=*[-0.024 0.009]. Models 4-7 evidenced no improved fit, *p*>.54. In the right dorsolateral prefrontal cortex, none of Models 1-6 depicted an improved fit in comparison to the null model, p>.14.

In the left temporo-parietal area, linear mixed-effects modeling revealed Model 2 and 3 to have improved model fit, *χ*^2^(2)=14.23, *p*=<.001, *χ*^2^(2)= 6.50, *p*=.038, respectively. Here, post-hoc tests reveal that trends in both earlier, *trend*=0.004, *SE*=0.006, 95% *CI=*[-0.007 0.017], and later epochs were both positive, *trend*=0.008, *SE*=0.006, 95% *CI=*[-0.004 0.021], and therefore showed no difference between epochs*.* Models 3-6 did not have additional explanatory power, *p*>.15. In the right temporo-parietal area, none of the Models 1-6 had a better model fit than the null model, p>.13.

Overall, the individual analyses reveal frontal and temporal areas in the left hemisphere to be involved in the effects of turn-taking with time.

1. HbR analyses

In addition to HbO values, HbR values were extracted and tested in the same approach as described for HbO values. In control analyses original dyads’ HbR coherences were tested against the average coherence value of 1,000 permutations of random pairs. However, original dyads did not show significantly higher coherences than the random pairs, *p*>.99. Still, we tested the same linear mixed models as assumed for HbO coherences (Models 1-7), which yielded no significant improvement of model fit from the Null Model, *p*>.47.

1. Communication patterns

Descriptives for communication patterns are reported in *SI Table 3*. Correlation analyses were run with the lm function and reported p-values are corrected for multiple comparisons with the Holm-Bonferroni method (Holm, 1979)*.* The correlation values of all four communication patterns are depicted in *SI* *Table 4.* Dyads with intrusive communication patterns had more relevant turns, *r*=.35, *p*<.01, but showed no other common pattern with contingent turns or turn-taking, *p*>.26. However, dyads who communicated in more relevant turns also showed more turn-taking, *r*=.60, *p*<.01, and contingency, *r*=.60, *p*<.01, in their communication patterns. In addition, turn-taking was highly correlated with more contingent answers, *r*=.64, *p*<.01.

1. Turn and overlap duration

During the four minutes of the conditions, mother-child dyads spoke for an average of 83.77% of the time (*SD*=4.25%, *range*=69.89-92.13%). On average, dyads conversed with switching pauses of 694 ms, ranging from 478 ms to 1224 ms. Out of 40 dyads, 36 dyads also had overlaps of verbalizations of an average of 415 ms (range=120-809 ms). Dyads showed high individual variation in the frequency of overlaps, averaging at 7 times during a four-minute free verbal conversation, but ranging from 0 to 35 times.

Supplementary tables

Table S1. Model Outputs (Model 1 - 7)

|  | *Estimates* | *SE* | *CI Lower* | *CI Upper* | *RE SD* | *X²* | *df* | *p* |
| --- | --- | --- | --- | --- | --- | --- | --- | --- |
| Model 1: *WTC ~ turn-taking + (1 + turn-taking \|\| dyad)* | | | | | | | | |
| Comparison to Nullmodel *WTC ~ 1 + (1\|dyad)* | | | | | | 6.83 | 2 | .033 |
| (Intercept) | -0.725 | 0.011 | -0.747 | -0.704 | 0.030 |  |  |  |
| turn-taking | 0.018 | 0.013 | -0.007 | 0.044 | 0.044 |  |  |  |
| Model 2: *WTC ~ turn-taking + time +*  *(1 + turn-taking + time \|\| dyad)* | | | | | | | | |
| Comparison to Model 1 | | | | | | 12.45 | 2 | .006 |
| (Intercept) | -0.755 | 0.018 | -0.790 | -0.721 | 0.000 |  |  |  |
| turn-taking | 0.011 | 0.015 | -0.018 | 0.040 | 0.047 |  |  |  |
| time | 0.007 | 0.004 | 0.006 | 0.018 | 0.010 |  |  |  |
| Model 3: *WTC ~ turn-taking + time + turn-taking : time +*  *(1 + turn-taking + time + turn-taking : time \|\| dyad)* | | | | | | | | |
| Comparison to Model 2 | | | | | | 19.54 | 2 | <.001 |
| (Intercept) | -0.755 | 0.018 | -0.748 | -0.704 | 0.000 |  |  |  |
| turn-taking | 0.018 | 0.013 | -0.007 | 0.045 | 0.044 |  |  |  |
| time | 0.012 | 0.010 | -0.008 | -0.008 | 0.028 |  |  |  |
| turn-taking:time | 0.016 | 0.013 | -0.009 | 0.013 | 0.042 |  |  |  |
| Model 4: *WTC ~ turn-taking + time + turn-taking : time + turn-taking duration +*  *(1 + turn-taking + time + turn-taking : time + turn-taking duration \|\| dyad)* | | | | | | | | |
| Comparison to Model 3 | | | | | | 2.40 | 2 | 0.301 |
|  | *Estimates* | *SE* | *CI Lower* | *CI Upper* | *RE SD* | *X²* | *df* | *p* |
| (Intercept) | -0.725 | 0.011 | -0.746 | -0.704 | 0.024 |  |  |  |
| turn-taking | 0.026 | 0.013 | 0.000 | 0.052 | 0.036 |  |  |  |
| time | 0.012 | 0.011 | -0.008 | 0.034 | 0.054 |  |  |  |
| turn-taking duration | 0.013 | 0.013 | -0.013 | 0.039 | 0.023 |  |  |  |
| turn-taking:time | 0.016 | 0.013 | -0.009 | 0.043 | 0.047 |  |  |  |
| Model 5: *WTC ~ turn-taking + time + turn-taking : time + relevance +*  *(1 + turn-taking + time + turn-taking : time + relevance \|\| dyad)* | | | | | | | | |
| Comparison to Model 3 | | | | | | 1.74 | 2 | 0.417 |
| (Intercept) | -0.725 | 0.011 | -0.745 | -0.704 | 0.018 |  |  |  |
| turn-taking | 0.018 | 0.015 | -0.012 | 0.048 | 0.029 |  |  |  |
| time | 0.013 | 0.011 | -0.009 | 0.034 | 0.028 |  |  |  |
| relevance | 0.006 | 0.019 | -0.031 | 0.042 | 0.041 |  |  |  |
| turn-taking:time | 0.017 | 0.013 | -0.010 | 0.043 | 0.047 |  |  |  |
| Model 6: *WTC ~ turn-taking + time + turn-taking : time + contingency +*  *(1 + turn-taking + time + turn-taking : time + contingency \|\| dyad)* | | | | | | | | |
|  |  |  |  |  |  | 0.37 | 2 | 0.832 |
| (Intercept) | -0.726 | 0.011 | -0.748 | -0.704 | 0.029 |  |  |  |
| turn-taking | 0.013 | 0.017 | -0.019 | 0.045 | 0.045 |  |  |  |
| time | 0.013 | 0.011 | -0.008 | 0.034 | 0.028 |  |  |  |
| contingency | 0.009 | 0.015 | -0.019 | 0.037 | 0.000 |  |  |  |
| turn-taking:time | 0.016 | 0.013 | -0.009 | 0.042 | 0.047 |  |  |  |
| Model 7: *WTC ~ turn-taking + time + turn-taking : time + intrusiveness +*  *(1 + turn-taking + time + turn-taking : time + intrusiveness \|\| dyad)* | | | | | | | | |
|  |  |  |  |  |  | 0.38 | 2 | 0.825 |
| (Intercept) | -0.726 | 0.011 | -0.748 | -0.704 | 0.019 |  |  |  |
| turn-taking | 0.019 | 0.013 | -0.006 | 0.045 | 0.045 |  |  |  |
| time | 0.012 | 0.010 | -0.008 | 0.034 | 0.028 |  |  |  |
| intrusiveness | 0.001 | 0.014 | -0.027 | 0.030 | 0.024 |  |  |  |
| turn-taking:time | 0.016 | 0.013 | -0.009 | 0.042 | 0.047 |  |  |  |

*Note.*

(1) The factor time was dummy coded and had the first time point as reference level. Estimates for the single predictors indicate the change from the response when the predictor changes from the reference level to the level of the predictor (in parentheses).

(2) Confidence intervals were derived using the Wald method using the function confint.

Included are Estimates, standard errors (SE), confidence intervals (CI), the standard deviation of the random effect (RE.SD) and likelihood ratio test outputs for the model comparisons.

Table S3. Descriptives of communication patterns including mean, standard deviation and range.

| Descriptives | | | | | | | | | |
| --- | --- | --- | --- | --- | --- | --- | --- | --- | --- |
|  |  |  |  |  |  |  |  |  |  |
|  | | **intrusive** | | **relevance** | | **turn-taking** | | **contingency** | |
| N |  | 40 |  | 40 |  | 40 |  | 40 |  |
| Missing |  | 0 |  | 0 |  | 0 |  | 0 |  |
| Mean |  | 3.92 |  | 66.5 |  | 39.5 |  | 17.5 |  |
| Standard deviation |  | 2.96 |  | 15.5 |  | 14.9 |  | 5.80 |  |
| Minimum |  | 0 |  | 27 |  | 14 |  | 3 |  |
| Maximum |  | 13 |  | 98 |  | 75 |  | 32 |  |
|  | | | | | | | | | |

Table S4. Correlation values of communication patterns (N=40).

| Correlation Matrix | | | | | | | | | | | | |
| --- | --- | --- | --- | --- | --- | --- | --- | --- | --- | --- | --- | --- |
|  |  |  |  |  |  |  |  |  |  |  |  |  |
|  | |  | | **intrusive** | | **relevance** | | **turn-taking** | | **contingency** | |  |
| intrusive |  | Pearson's r |  | — |  |  |  |  |  |  |  |  |
|  |  | p-value |  | — |  |  |  |  |  |  |  |  |
| relevance |  | Pearson's r |  | 0.347 | * | — |  |  |  |  |  |  |
|  |  | p-value |  | 0.028 |  | — |  |  |  |  |  |  |
| turn-taking |  | Pearson's r |  | -0.181 |  | 0.598 | *** | — |  |  |  |  |
|  |  | p-value |  | 0.265 |  | < .001 |  | — |  |  |  |  |
| contingency |  | Pearson's r |  | 0.002 |  | 0.602 | *** | 0.637 | *** | — |  |  |
|  |  | p-value |  | 0.990 |  | < .001 |  | < .001 |  | — |  |  |
| Note. * p < .05, ** p < .01, *** p < .001 | | | | | | | | | | | | |

*Supplementary Figure*


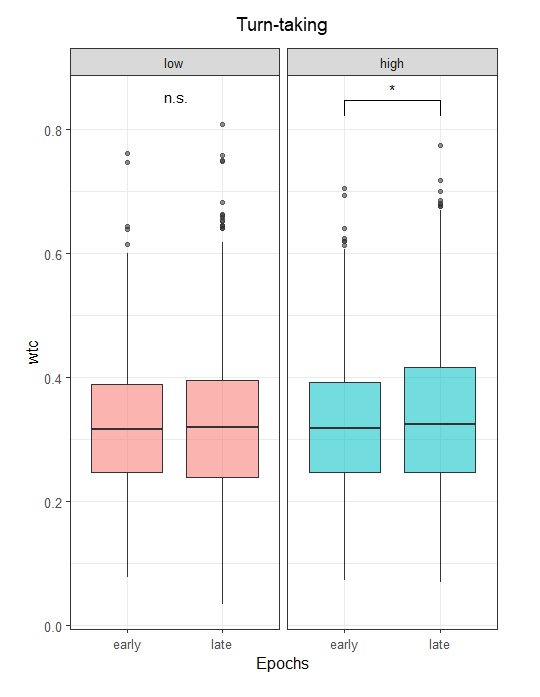


Figure S1. The figure shows the increase in neural synchrony (indicated by wtc on the y-Axis) from early to late epochs (x-Axis) in the high turn-taking groups (right facet), while the low turn-taking group (left facet) shows no difference in neural synchrony from early to late epochs.

Bibliography

Lerner, Y., Honey, C. J., Silbert, L. J., & Hasson, U. (2011). Topographic mapping of a hierarchy of temporal receptive windows using a narrated story. The Journal of Neuroscience: The Official Journal of the Society for Neuroscience, 31(8), 2906–2915.<https://doi.org/10.1523/JNEUROSCI.3684-10.2011>
